# Lack of Patient Knowledge Regarding the Adverse Effects of Analgesics With High Doses Leading to Elevation of Creatinine and Major Consequences – A Case Report

**DOI:** 10.7759/cureus.10891

**Published:** 2020-10-10

**Authors:** Krishna Teja Challa, Sateesh Babu Arja, Mirela Ponduchi, Baby M Snigdha

**Affiliations:** 1 Department of Medicine and Reserach, Avalon University School of Medicine, Willemstad, CUW; 2 Medicine / Clinical Skills, Avalon University School of Medicine, Willemstad, CUW; 3 Department of Internal Medicine, Phoenix St. Luke's Hospital, Phoenix, USA; 4 Internal Medicine, Mountain Vista Medical Center, Mesa, USA; 5 Internal Medicine, St. James School of Medicine, Arnos Vale, VCT

**Keywords:** analgesics, nsaids, dialysis, interstitial nephritis, creatinine

## Abstract

We present a case of a 49-year-old male with complaints of back pain and not being able to urinate. The patient was suffering from back pain for the last four days and followed up with the chiropractor, but the pain persisted. The patient took eight ibuprofen tablets (1600 mg) within those four days to relieve the pain. Lab workup showed a blood urea nitrogen (BUN) of 175 mg/dL, creatinine level of 32.87mg/dL, and an anion gap metabolic acidosis. With close monitoring and dialysis in the hospital, the creatinine came down to 11.92mg/dL. Ultrasound-guided renal biopsy showed that the patient developed acute interstitial nephritis. The patient was treated with prednisone and later discharged with a creatinine level of 8.60mg/dL. Before he was discharged, he was declared to have end-stage renal disease and placed on outpatient dialysis. Only a few case reports are recorded in the literature with such a high elevation of creatinine levels.

## Introduction

With an estimated daily usage of >30 million doses, nonsteroidal anti-inflammatory drugs (NSAIDs) are among the most common classes of medication used worldwide. NSAIDs work by suppressing prostaglandin (PG) synthesis, which then inhibits the enzyme cyclooxygenase (COX) and exerts its analgesic, antipyretic, and anti-inflammatory effects. The NSAIDs are known to have damaging effects on the kidneys and gastrointestinal tract when taken in large doses. Approximately 2.5 million Americans have been reported to have NSAIDs-mediated renal damage yearly [1]. There have been more than 70 million prescriptions written annually for NSAIDs in the United States alone, and they are also bought over the counter. It was estimated that more than 29 million US adults were using NSAIDs regularly in 2010, which was a 41% increase from 2005. A self-reported study of over-the-counter and prescribed use of ibuprofen, which falls into the class of NSAIDs, noted that 90% took it regularly. In comparison, 37% took other forms of NSAIDs in addition to ibuprofen, and 11% exceeded the daily recommended limit of ibuprofen [2]. This case report emphasizes the importance of public awareness regarding analgesics and its associated side effects with large doses.

## Case presentation

A 49-year-old male patient without any significant past medical history presented to our hospital with problems of not voiding his bladder and back pain. The patient reported his back pain began four days ago when he was at work and tried to lift a 100 lb instrument. The patient went to see a chiropractor, but there was no improvement with his back pain. He then started to experience left thigh and buttock pain along with the back pain for which he took eight ibuprofen tablets a day (1600 mg) for four days before presenting to the hospital. He denies taking ibuprofen for any other pain that he experienced in the past.

While in the emergency room, the initial labs revealed creatinine of 32mg/dL, BUN of 175mg/dL, Na of 128 mEq/L, K of 4.5mmol/L, Cl of 90mEq/L, Co2 of 17.0mEq/L, anion gap of 21, Ca of 7.9mEq/L, and a total creatine kinase of 1315 micromoles/L. His liver function tests (LFTs) were also elevated - serum aspartate aminotransferase (AST) was 104 units, and alanine aminotransferase (ALT) was 281 units. His urinalysis showed 3+ blood and 2+ protein but 0-5 red blood cells. Left lower extremity Doppler ultrasound was negative for deep vein thrombosis (DVT). CT scan of the abdomen was unremarkable. There was fatty infiltration of the liver and thickening of the bladder wall, and diffuse soft tissue edema involving the left hip. The patient was given ondansetron hydrochloride (Zofran® Injection) 4 mg intravenously (IV) Q6H PRN for nausea, oxycodone hydrochloride (Roxicodone®) 5 mg PO Q4H PRN for pain, morphine sulfate 2 mg IV Q2H PRN for severe back pain, hydralazine hydrochloride (Apresoline® Injection) 10 mg IV Q4H PRN for hypertension, ceftriaxone sodium 1 gm/ (sodium chloride) 100 mls at 200 mls/hr IV Q24H at 1900 SCH for cystitis, and sodium bicarbonate 150 meq/ (dextrose) 1,150 mls at 150 mls/hr IV, Q7H40M SCH. Acute kidney injury and metabolic acidosis was the impression on evaluation in the ED.

Later the patient was moved to the telemetry unit to be followed up with the hospitalist. The patient was monitored by an internist, and a nephrology consultation was requested. On the following day, during the nephrology consultation, the patient's laboratory values were as shown in Tables 1-3. The patient's vitals are mentioned in Table 4. CT scan and renal ultrasound of the kidneys were ordered. CT scan of the abdomen was unremarkable. The nephrologist's recommendation was to continue the patient on the IV fluids with bicarbonate and avoid nephrotoxins. The nephrologist closely monitored the patient, and the plan was to place the patient on temporary dialysis if the kidney function doesn't show improvement. 

**Table 1:** Patient's hematologic laboratory findings WBC - white blood cells; RBC - red blood cells; Hbg - hemoglobin; Hct - hematocrit; MCV - mean corpuscular volume; MCH - mean corpuscular hemoglobin; MCHC - mean corpuscular hemoglobin concentration; RDW - red cell distribution width; BUN - blood urea nitrogen; CK - creatine kinase; Est GFR - estimated glomerular filtration rate; AST - serum aspartate aminotransferase; ALT - alanine aminotransferase; AIN - acute interstitial nephritis

Labs	On admission	Next day	1^st^ round of hemodialysis	4^th^ round of hemodialysis	Diagnosis of AIN	7^th^ round of hemodialysis	On the day of discharge
WBC	9.0	11.7	14.0	11.8	12.1	11.8	12.9
RBC	4.38	3.18	3.43	3.43	3.09	3.05	3.09
Hbg	14.2	13.6	10.1	11.0	9.9	9.7	9.9
Hct	39.3	28.4	29.6	31.1	28.5	27.8	28.5
MCV	89.7	88.8	93.1	90.7	92.0	91.1	92.2
MCH	32.4	31.7	32.0	31.8	32.0	31.5	32.0
MCHC	36.1	34.1	35.4	34.9	34.7	34.9	34.7
RDW	13.4	12.3	12.4	12.3	12.0	12.0	12.0
Na	128	128	133	134	133	135	137
K	4.5	4.1	4.5	4.2	4.7	4.7	4.4
Cl	90	88	96	98	97	97	100
Co2	17.0	18.0	28.0	24.0	27.0	28.0	28.0
Anion gap	21	22	9	8	9	8	9
BUN (mg/dL)	175	171	60	37	39	37	18
Creatinine (mg/dL)	32.87	30.2	18.60	11.92	14.29	10.80	8.60
Ca	7.9	7.3	7.7	7.9	8.7	8.7	8.9
Phosphorus	11.60	11.6	8.9	9.5	9.0	9.5	7.0
CK	1315	-	-	-	-	-	300
Est GFR (Afr Amer)	2	2	5	5	4	6	8
Est GFR (Non-Afr Amer)	2	2	4	4	4	5	7
AST	25	24	22	17	17	16	16
ALT	90	85	65	54	49	45	46
BUN/ Cr	5.32	5.6	4.7	3.10	2.72	3.42	2

**Table 2:** Patient's urine findings RBC - red blood cells; WBC - white blood cells

Labs	On admission	On the 7^th^ round of hemodialysis	On the day of discharge
Urine color	Yellow	Yellow	Yellow
Urine clarity	Clear	Clear	Clear
Urine PH	6.0	6.0	6.0
Urine specific gravity	1.020	1.010	1.010
Urine protein	2+	2+	2+
Urine glucose	Trace	Trace	Trace
Urine ketones	Negative	Negative	Negative
Urine squamous epithelial cells	Rare	Rare	Rare
Urine RBC	Rare	Rare	Rare
Urine WBC	5-10	5-10	2-5

**Table 3:** Patient's immunology findings ANCA - antineutrophil cytoplasmic antibodies; C-ANCA - cytoplasmic antineutrophil cytoplasmic antibodies; P-ANCA - perinuclear antineutrophil cytoplasmic antibody

Labs	
ANCA reference labs	Negative
C-ANCA antibody	<1:20
Proteinase 3 (PR3) antibodies	<3.5
Atypical P-ANCA	<1:20
P-ANCA antibody	<1:20
Anti-myeloperoxidase	<9.0
Glomerular basement membrane antibody	Pending

**Table 4:** Patient's vitals

Vitals	Emergency room	In telemetry unit	At the time of discharge
Temperate	36.4 C	36.6 C	36.6 C
Pulse	65/min	60/min	68/min
Blood pressure	142/68 mm of hg	132/78 mm of hg	130/70 mm of hg
Respiratory rate	18/min	16/min	17/min
Pulse oximetry	94% sat	98% sat	98% sat

On a subsequent day on follow up with the internist, the kidney function tests were not improving; upon discussion with the nephrologist, a temporary dialysis catheter order was placed. On the same day, an interventional radiologist placed a right internal jugular vein (IJV) temporary dialysis catheter, and the patient went for the first round of hemodialysis treatment, after which the creatinine level went down to 18 mg/dl. Urinalysis was positive for protein and red blood cells, and the labs of creatine phosphokinase (CPK) 1315 micromoles/L pointed towards rhabdomyolysis. The next day during the nephrology consultation, it was recommended to go for the second round of hemodialysis. The patient's lab showed phosphorus of 11.60 mg/dL, leading to sevelamer 800 mg PO TID initiation. The patient underwent another four rounds of hemodialysis, and the kidney function tests showed a creatinine level of 11.92 mg/dL.

**Figure 1:**
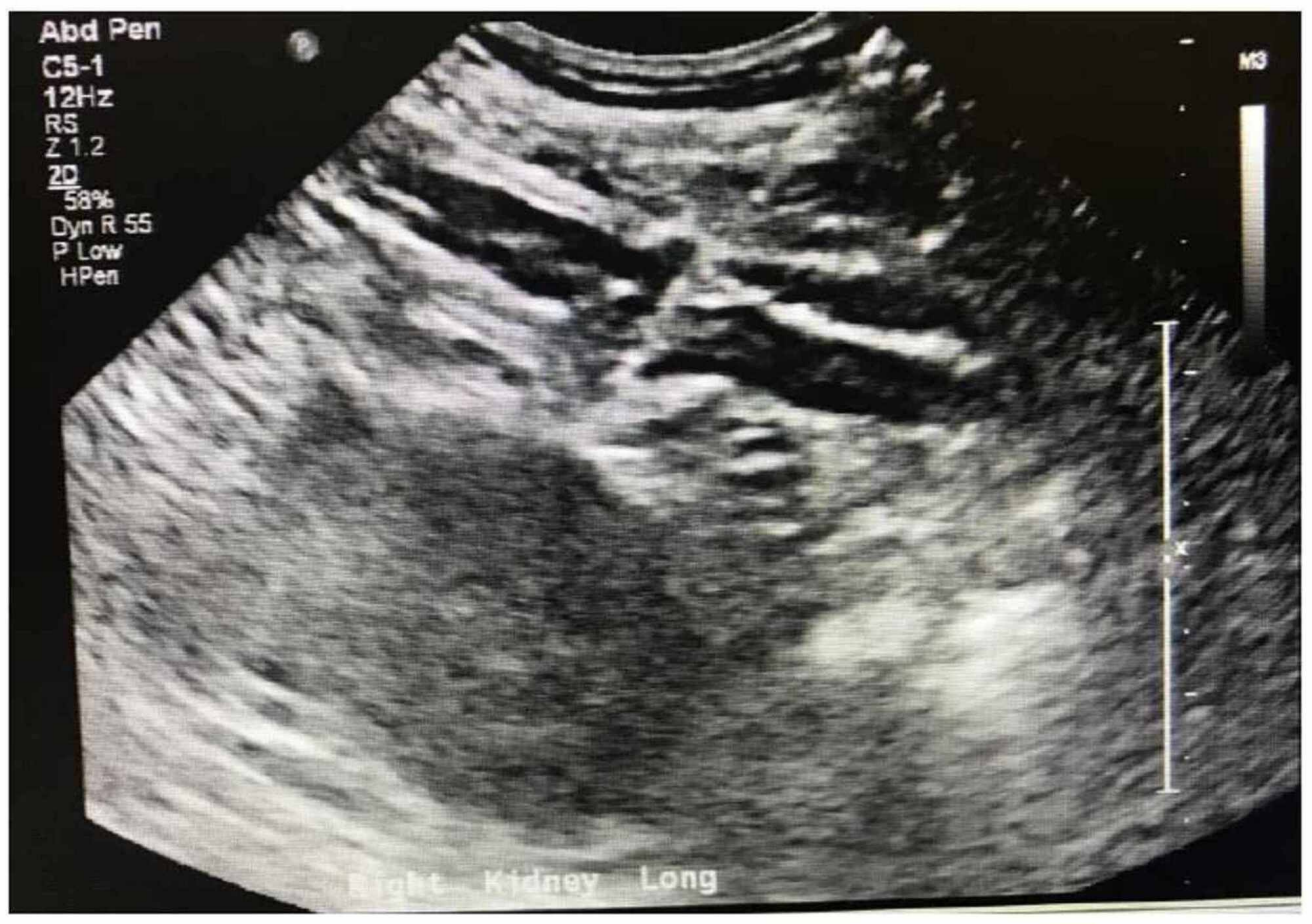
Ultrasound-guided needle biopsy of the right kidney

The patient subsequently went through hemodialysis for the seventh time, but his kidney function had not yet returned to the normal level. The nephrologist ordered a tunneled dialysis catheter to be placed on the patient to be able to receive outpatient dialysis once he is discharged, which was also placed by an interventional radiologist. His labs were checked again, and the results showed that his CPK, hyponatremia, and hyperkalemia were resolving to normal, but the creatinine stayed stable at a high level of 10.6 mg/dL. Due to the lack of improvement in the kidney function tests towards normal range, the nephrologist ordered a renal biopsy. A day later, a kidney biopsy was done (Figure 1), which showed acute interstitial nephritis with very minimal chronicity, and the patient was started on prednisone 60 mg once a day. Because of the lack of improvement on the kidney function test to normal levels, the patient was declared to have end-stage renal disease and was discharged with the outpatient dialysis treatment program and to follow up with the nephrologist.

## Discussion

Creatinine levels are used to assess the kidney's functioning, along with the glomerular filtration rate (GFR) and BUN. As creatinine is a waste product of our muscles, its levels are dependent upon the person's age, gender, and race, but average creatinine levels should be between 0.6-1.3 mg/dL. Acute kidney injury occurs when there is damage to the kidney within a few hours or days. As a result, waste products start building up in our body, which can then be measured by the serum creatinine level. There were previously only four reported cases on creatinine levels being too high [3], and with our patient, we are able to add another case.

Studies have shown that NSAIDs' initiation was associated with an increased number of hospitalizations with acute kidney failure [4]. They cause acute kidney injury in patients who take them when they suffer from acute or chronic pain. Many people are not aware of the side effects of NSAIDs can have on the body, especially the kidneys, and the dose limit at which they can consume it. Because NSAIDs are freely available over-the-counter, these drugs' adverse effects are unregulated and underrecognized as drug agents that are potentially dangerous to take at large doses [4]. This was the case of our patient, who unknowingly took 1600 mg of ibuprofen, which led to his kidney damage.

By the time our patient found out something was wrong because he could not urinate, it was too late for him as damage to his kidney already occurred. He was seen by the nephrologist who recommended hemodialysis in the hospital to see if his creatinine can be brought back down to a near-normal level, which failed after hemodialysis courses, and the patient was eventually sent home with regular visits to the nephrologist for outpatient dialysis. Due to the lack of knowledge on the proper dose of NSAIDs use, action has been taken over the recent years to spread discussion about the serious matter at hand. Nonpharmacologic and pharmacological interventions other than NSAIDs have been emphasized when dealing with pain [5]. Our case highlights the need for the importance of public awareness regarding the adverse effects associated with the use of NSAIDs, especially high doses of NSAIDs.

## Conclusions

Many over-the-counter (OTC) medications are readily available for people to purchase when they suffer from mild illnesses without a physician's need for a prescription. NSAIDs are among those readily available OTC medications that can relieve mild pain and cause kidney injury when taken in large doses. Our case report emphasizes the need for patient education regarding the adverse effects associated with the use of high doses of NSAIDs and the importance of finding innovative research on different treatment options for pain, such as other pharmacological and nonpharmacological treatments.
